# The Gut Microbiota from Lean and Obese Subjects Contribute Differently to the Fermentation of Arabinogalactan and Inulin

**DOI:** 10.1371/journal.pone.0159236

**Published:** 2016-07-13

**Authors:** Marisol Aguirre, Carlota Bussolo de Souza, Koen Venema

**Affiliations:** 1 Top Institute of Food & Nutrition (TIFN), Wageningen, The Netherlands; 2 Maastricht University, School of Nutrition and Translational Research in Metabolism (NUTRIM), Faculty of Health, Medicine and Life Sciences, Department of Human Biology, Maastricht, The Netherlands; 3 The Netherlands Organization for Applied Scientific research (TNO), Zeist, The Netherlands; 4 CNPq, Brasilia, Brazil; 5 University of Groningen, Groningen, The Netherlands; 6 Beneficial Microbes Consultancy, Wageningen, The Netherlands; Max Rubner-Institut, GERMANY

## Abstract

**Background:**

An aberrant metabolic activity or a compositional alteration of the gut microbiota has been proposed as a factor that makes us more prone to disease. Therefore, we explored the effect of two dietary fibers (arabinogalactan and inulin) on the microbiota from lean and obese subjects during 72 h *in vitro* fermentation experiments using the validated TNO dynamic *in vitro* model of the proximal colon: TIM-2. Metabolically, arabinogalactan fermentation showed a higher production of propionate when compared to *n*-butyrate in the obese microbiota fermentations. In general, lean microbiota produced more *n*-butyrate from the fermentation of both substrates when compared to the obese microbiota. Furthermore, the obese microbiota extracted more energy from the fermentation of both fibers.

**Results:**

Compositionally, bacteria belonging to *Gemmiger, Dorea, Roseburia, Alistipes, Lactobacillus* and *Bifidobacterium* genera were found to be highly abundant or stimulated by the prebiotics in the lean microbiota suggesting a potential role in leanness. Furthermore, a significant correlation between known butyrogenic strains including *B. adolescentis*, an unclassified *Bifidobacterium* and *F. prausnitzii* with this metabolite in the fermentation of inulin in both microbiotas was found.

**Conclusions:**

Although supplementary *in vivo* studies are needed, the current study provides more evidence for the consumption of specific ingredients with the aim of modulating the gut microbiota in the context of obesity.

## Introduction

The discovery of the potential impact of the gut microbiota on human health and disease has fuelled research on characterizing the role that this community plays in the causality or prevention of many diseases elicited by dangerous lifestyles such as sedentary and bad eating habits, among others [1, 2].

Part of the efforts have been focused on identifying a balanced and thus, healthy community [3]. Though provocative, it is difficult to define a “most desirable” composition for the human gut microbiota. Reports providing contradictory findings, due to either i) a large inter-individual variation or ii) the application of different analytic methods, are at the order of the day. However, another factor which seems to play an important role in influencing health and disease, besides the community composition, is the interaction of the microbial metabolites with the host.

The fermentation of dietary fiber by the gut microbiota leads primarily to the production of short-chain fatty acids (SCFA; mainly acetate, propionate and butyrate) and the gases hydrogen, methane and carbon dioxide [4]. Furthermore, branched-chain fatty acids (BCFA; mainly *iso*-butyrate and *iso*-valerate often accompanied by phenol and ammonia production) are also produced to a lesser extent but these mostly originate from protein fermentation [5]. A proposed mechanism by which fiber may protect us against obesity is based on the beneficial effects that such metabolites have on host energy balance, e.g. by mediating the secretion of gut hormones involved in the regulation of energy metabolism and food intake (including leptin, peptide YY and glucagon-like peptide-1) [6, 7]. Thus, it may be tempting to say that high intake of fiber would be a way to reduce the risk of obesity [8, 9]. After all, it is estimated that the production of SCFA by the microbiota accounts for 5 to 10% of total dietary energy requirements in humans [10]. However, recent research has questioned such risk-reduction role. There is growing evidence indicating that the production of SCFA differs between the microbiota originating from obese and lean individuals (hereafter referred to as obese and lean microbiota). Such difference lies in the fact that the obese microbiota may produce more SCFA which could be translated into more energy extraction from diet [11, 12]. As a consequence, more energy extracted from diet may be stored as fat, promoting host’s weight gain. Such mechanisms place fiber fermentation by the gut microbiota as a causative factor in obesity. Still, as previously remarked, there is a lack of consistency, different studies show contrasting results by finding either no correlation between fiber and weight gain/obesity, a reverse trend or effects to be substrate dependent [12–14].

Vast amounts of research are needed to answer the chicken or the egg causality dilemma before any strategy can be designed with the aim of manipulating the gut microbiota in the context of obesity. Currently, there is a limited number of *in vitro* fermentation experiments mimicking the fermentation of different substrates by human obese or lean microbiota. So far, these studies have provided evidence about the metabolic adaptation of the microbiota in relation to different nutrient loads or single testing of specific prebiotics, as well as the plasticity of the microbiota in configuring the structure of the community in response to these kind of interventions [14–19]. Importantly, these studies have also endorsed *in vitro* systems as tools facilitating the medium to high-throughput validation of multiple hypotheses at lower costs with no ethical constraints when compared to human or animal studies.

The purpose of the current study was to compare the profiles of fermentation of arabinogalactan (AG) and the well-studied prebiotic inulin (IN) by obese or lean microbiota. Both AG and IN are natural polysaccharides commonly found in foods. They have been found to be fermented by human intestinal bacteria and stimulate the production of SCFA and the growth of specific bacteria generally believed to be beneficial to the host [20–22]. AG is an interesting compound to evaluate not only because its potential to improve gut barrier function [23] but also because it has been observed that it may induce production of (both pro and anti-inflammatory) cytokines [24, 25], factors which both may play an important role in inflammation. In the context of obesity, low grade inflammation has been suggested to contribute to the development of insulin and leptin resistance [26].

In order to compare the two prebiotics, we performed 72h fermentation experiments in the validated TNO dynamic *in vitro* model of the proximal colon (TIM-2), which was inoculated with either obese or lean microbiota. The present work brings evidence about how fermentable carbohydrates are differently used by the microbiota from lean and obese subjects which contributes to the understanding on how dietary compounds could be used as therapeutic tools in obesity.

## Materials and Methods

### Gut microbiota

The inocula used for the TIM-2 experiments consisted of an active, pooled fecal microbiota prepared from: i) 8 healthy lean volunteers (male: n = 4, female: n = 4, average age = 31 y (range: 25–42), BMI = 20 ± 1.48 kg/m^2^); ii) 7 healthy obese volunteers (male: n = 3, female: n = 4, average age = 51 y (range: 29–68), BMI = 32 ± 1.17 kg/m^2^. We have previously shown that pooling does not result in an aberrant microbiota composition or activity [27]. The exclusion criteria for lean and obese volunteers included the use of antibiotics during the preceding 3 months, gastrointestinal disease, severe chronic disease or food allergy and intake of probiotics and prebiotics.

Whole fecal samples were self-collected in a container kit which was maintained under anaerobiosis by using anaerobic packs (AnaeroGen^™^, Oxoid, Cambridge, UK). A sample aliquot (100 mg) from each individual donation was collected in an anaerobic cabinet (80% N_2_, 10% CO_2_, 10% H_2_), snap-frozen in liquid nitrogen (-196°C) and stored at -80°C for measurement of metabolites (SCFA and BCFA). Feces were homogenized under anaerobic conditions as described by Aguirre *et al*. [28]. The resulting culture homogenate was aliquoted and snap-frozen in liquid nitrogen. This microbiota was stored at -80°C before inoculation in TIM-2.

### Gut fermentation experiments

The TIM-2 system was flushed with N_2_ prior to the introduction of the inoculum for 3 h and it was maintained under this condition at 37°C for 96 h with the pH kept at or above 5.8 by automatic titration with 2M NaOH. A 30 ml portion of culture homogenate was used to inoculate the units for each experiment. The microbiota was left to adapt (16 h) to the new environment after inoculation and during this period the basal simulated ileal efflux medium (SIEM) was gradually introduced into the system in a total volume of 40 ml. After the adaptation, the culture was deprived from any medium for 2 h (starvation). A volume of 180 ml of the different diets and control was administrated over the 72 h of the test period at a rate of 2.5 ml/h.

In order to remove water and fermentation products from the lumen, a dialysate system (described in detail by van Nuenen *et al*. [29]), consisting of a semi-permeable hollow membrane, ran through the lumen. For all the experiments, the speed of the dialysis fluid was set at 1.5 ml/ min.

After 24 and 48 h of fermentation 25 ml of lumen sample was removed from the system to mimic the transit of material from the proximal and reaching the distal colon [30]. Luminal and dialysate samples were taken after t = 0, 24, 48 and 72 h. In all cases samples were snap-frozen in liquid nitrogen and stored (-80°C) until analysis.

### Fermentation media

During the adaptation period (16 h) all TIM-2 units were fed with SIEM as described by Maathuis *et al*.[31]. After this adaptation period and the 2h starvation period, the units were fed with preparations which were made containing approximately 7.5 g of AG or IN instead of the standard carbohydrates in SIEM. The specific AG used in this study was (+)-Arabinogalactan- from larch wood (Sigma-Aldrich, St Louis, USA) with a molecular weight ranging from 72–92 kDa and ≥ 84.8% purity. The IN tested had degree of polymerization (DP) of 9; 84.9% > DP5 (Sensus, Frutafit^®^ IQ, Roosendaal, The Netherlands). Control experiments were performed in parallel to the experiments testing either AG or IN. SIEM was used to feed the microbiota in such controls.

### Analysis of SCFA (acetate, propionate, and *n*-butyrate) and BCFA (*iso*-butyrate and *iso*-valerate)

Samples were prepared and analyzed as described previously [14]. Before centrifuging, the fecal aliquots from the individuals were suspended in PBS (1:1; w:w). Briefly, both suspended aliquots and TIM-2 luminal samples were centrifuged (12000 r.p.m at 4°C for 10 min). To the clear supernatant a mixture of formic acid (20%), methanol and 2-ethyl butyric acid (internal standard, 2 mg/ml in methanol) was added. A 3 μl sample with a split ratio of 75.0 was injected on a GC-column (ZB-5HT inferno, ID 0.52 mm, film thickness 0.10 um; Zebron; phenomenex, USA) in a Shimadzu GC-2014 gas chromatograph. Standard curves were obtained by injecting calibrated quantities of a blend of volatile fatty acids and amounts were calculated from the graph obtained correlating peak height and time measured (all reagents from Sigma-Aldrich with the exception of formic acid which was from Merck).

### Energy extraction

Energy extraction in the form of SCFA was calculated using the following kJ mol^-1^ values for acetate, propionate and *n*-butyrate respectively: 874, 1536 and 2192 [32, 33].

### Characterization of bacterial populations

RNA was isolated from luminal samples using standard molecular biology kits from ZYMO Research (Zymo Research Co., CA, USA) following manufacturer’s instructions. Reverse-transcriptase amplification of the 16S rRNA gene (V3-V4), barcoding and library preparation (1st step PCR and 2nd PCR) were performed by BaseClear, Leiden, The Netherlands.

Short paired-end sequence reads were generated using the Illumina MiSeq system and converted into FASTQ files using the BCL2FASTQ pipeline version 1.8.3. Quality trimming was applied based on Phred quality scores. Subsequently, the Illumina paired reads were merged into single reads (so-called pseudoreads) through sequence overlap (16S rRNA V3V4 region of about 500bp). Chimeric pseudoreads were removed and the remaining reads were aligned to a combination of the GreenGenes and RDP 16S gene databases [34, 35]. Based on the alignment scores of the pseudoreads, the taxonomic classes were assigned by associating each pseudoread to the best matching Operational Taxonomic Unit (OTU). The taxonomic depth of the lineage is based on the identity threshold of the rank; Species 99%, Genus 97%, Family 95%, Order 90%, Class 85%, Phylum 80%.

### Data analysis

The experiments were performed in series of two per tested substrate (n = 2). These replicates were conducted for each microbiota (*i*.*e*. lean or obese). To avoid unnecessary repetition, this is not indicated further in the text or graphs in the results section. Results are displayed as average of these duplicates. For simplicity of reading, substrates in the following sections are tagged with the letter L or O (e.g. substrate-L, substrate-O) in order to refer to the fermentation experiments using the inoculum from lean (-L) or obese (-O) subjects.

Statistical analyses for determining the differences in metabolite production and energy extraction in the fecal samples from each individual were performed (SPSS for Windows, version 21, SPSS, Chicago, US). Comparison between the two groups (lean and obese) was performed using t-test with significance p<0.05.

For the calculation of fold compositional changes the ratio between a sampling time point and t0 was calculated (i.e., t72/t0). Then the ratio for this value and the control was then determined to obtain fold changes. A value equal to 1 indicates no change; a value of >1 indicates an increase; and a value of <1 indicates a decrease of the respective microbial genera.

A correlation analysis was performed in order to test if the metabolites measured would positively or negatively correlate with the different bacterial groups fed either with AG or IN. To these means, differentially abundant bacterial species growing on the different tested substrates were calculated from a ratio based from the specific growth of the species found on each substrate and the control. Spearman correlations were calculated between the ratio of the species identified as being differentially abundant and the measured amounts of metabolites produced (SPSS for Windows, version 21, SPSS, Chicago, US). Correlations were considered significant at the 0.01 level (2-tailed).

### Ethics Statement

Studies using fecal donations from healthy volunteers do not require medical ethical committee approval in The Netherlands since they are considered as non-invasive. However, volunteers who donated the inoculum were informed prior to initiating the study and their participation was considered after providing a signed informed consent. The group of obese donors were recruited at Maastricht University Medical Center (The Netherlands). These were patients from the university medical center who voluntarily responded to a recruiting call from inviting to donate their feces. The group of lean donors were recruited at TNO (The Netherlands). These participants responded to an advertisement inviting subjects to voluntarily collect their feces. Bouke Salden and Carlota Bussolo de Souza personally collected the fecal samples from the participants who exclusively donated their feces for the present study. Bouke Salden received the fecal samples directly from the obese participants and none of the authors was involved in the direct collection of these samples. Carlota Bussolo de Souza received the fecal samples directly from the lean participants. The origins of the both lean and obese fecal donations were blinded using a code whose identity was known only by the responsible scientist (Carlota Bussolo de Souza, co-author of the present study). Results in this manuscript are referred to an individual sample or a pool fecal inoculum and do not directly refer to a particular person.

## Results

### Screening of metabolites and energy extraction in feces from the volunteers

The average amount of SCFA found in the feces from lean and obese subjects were not statistically different (259.6 ± 100.2 mmol and 215.1 ± 66.9 mmol, respectively). However, it was clear that there was a great inter-individual difference regarding the amount of each SCFA in both groups (S1 Table). When comparing the production of each SCFA and the amount of energy extracted, no statistically significant differences were found.

BCFA were higher in the feces from lean volunteers (p<0.05). On average, lean subjects produced 7.88 ± 2.54 mmol of *iso*-butyrate and 11.45 ± 3.64 mmol of *i*-valerate, while obese subjects produced on average 3.11 ± 1.98 mmol of *i*-butyrate and 4.85 ± 2.92 mmol of *i*-valerate. BCFA production also presented a great inter-individual difference among subjects.

### Fermentation experiments with lean and obese microbiota

#### Microbial activity

**Fig 1:**
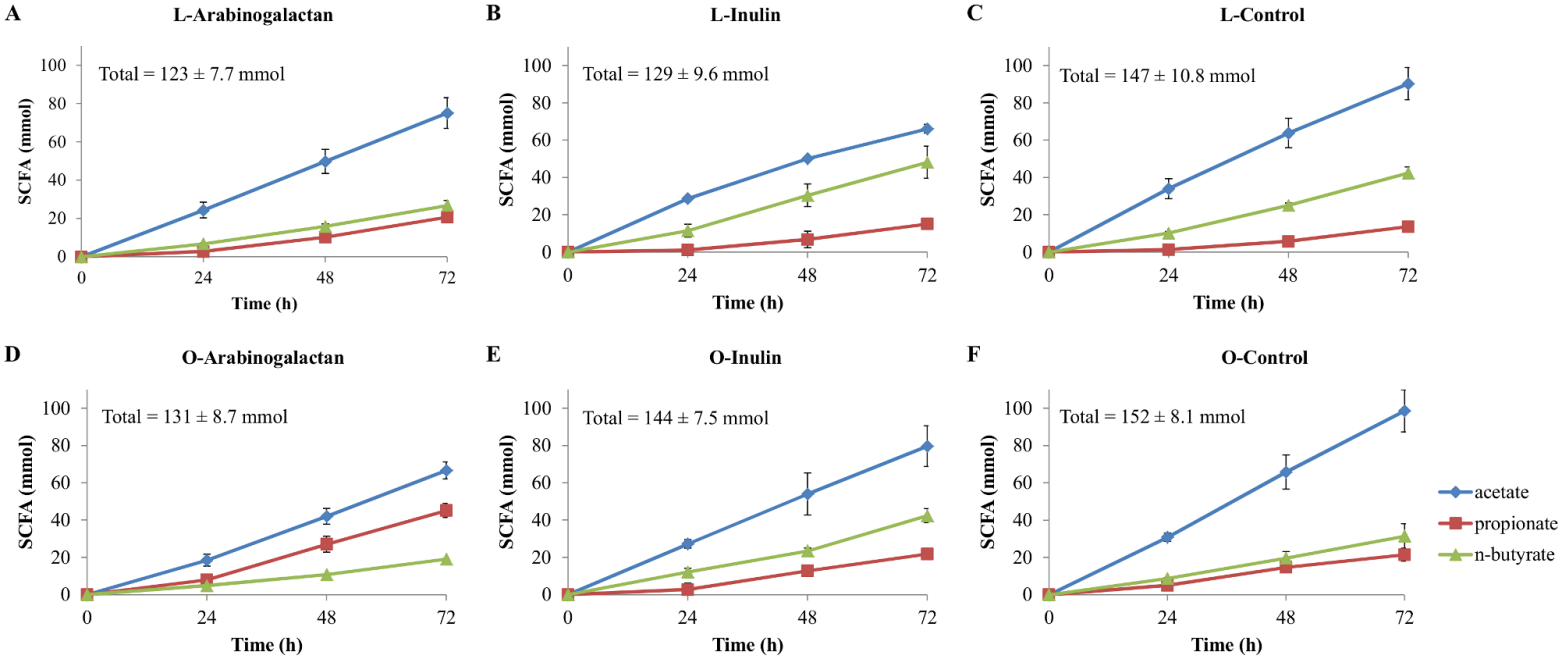
Cumulative production of SCFA (mmol) during the 72 h of fermentation of the tested substrates.

Total SCFA production was higher in fermentations using the obese microbiota when compared to the lean (Fig 1). Fermentation kinetics in terms of SCFA production observed from both inocula differed in AG, IN and control experiments. Fermentation of AG showed major differences in propionate and *n*-butyrate production compared to the other fermentations, with propionate even higher than *n*-butyrate in the obese microbiota fermentations. In general, *n*-butyrate production is higher in the lean fermentations for all substrates when compared to obese, while propionate is observed to be higher in the fermentations with the obese microbiota when compared to lean.

Table 1 shows the cumulative production of BCFA after 72 h of fermentation of AG, IN and control. Lean microbiota produced more BCFA from AG fermentation and less from IN than control. For AG this was also observed in the obese microbiota. The values from the obese fermentations were lower when compared to lean for control and AG, but not for IN.

**Table 1:** Cumulative production of BCFA after 72 h of fermentation of AG, IN and control.

Test	Lean			Obese		
Compound	*i*-butyrate	*i*-valerate	Total	*i*-butyrate	*i*-valerate	Total
Arabinogalactan	1.62 ± 0.66	2.32 ± 0.08	3.94 ± 0.75	0.55 ± 0.36	1.59 ± 0.38	2.13 ± 0.74
Inulin	0.22 ± 0.2	1.23 ± 0.15	1.45 ± 0.35	0.45 ± 0.07	1.49 ± 0.08	1.94 ± 0.15
Control	0.74 ± 0.14	1.99 ± 0.4	2.73 ± 0.27	0.44 ± 0.28	1.22 ± 0.06	1.66 ± 0.22

#### Energy extraction

**Fig 2:**
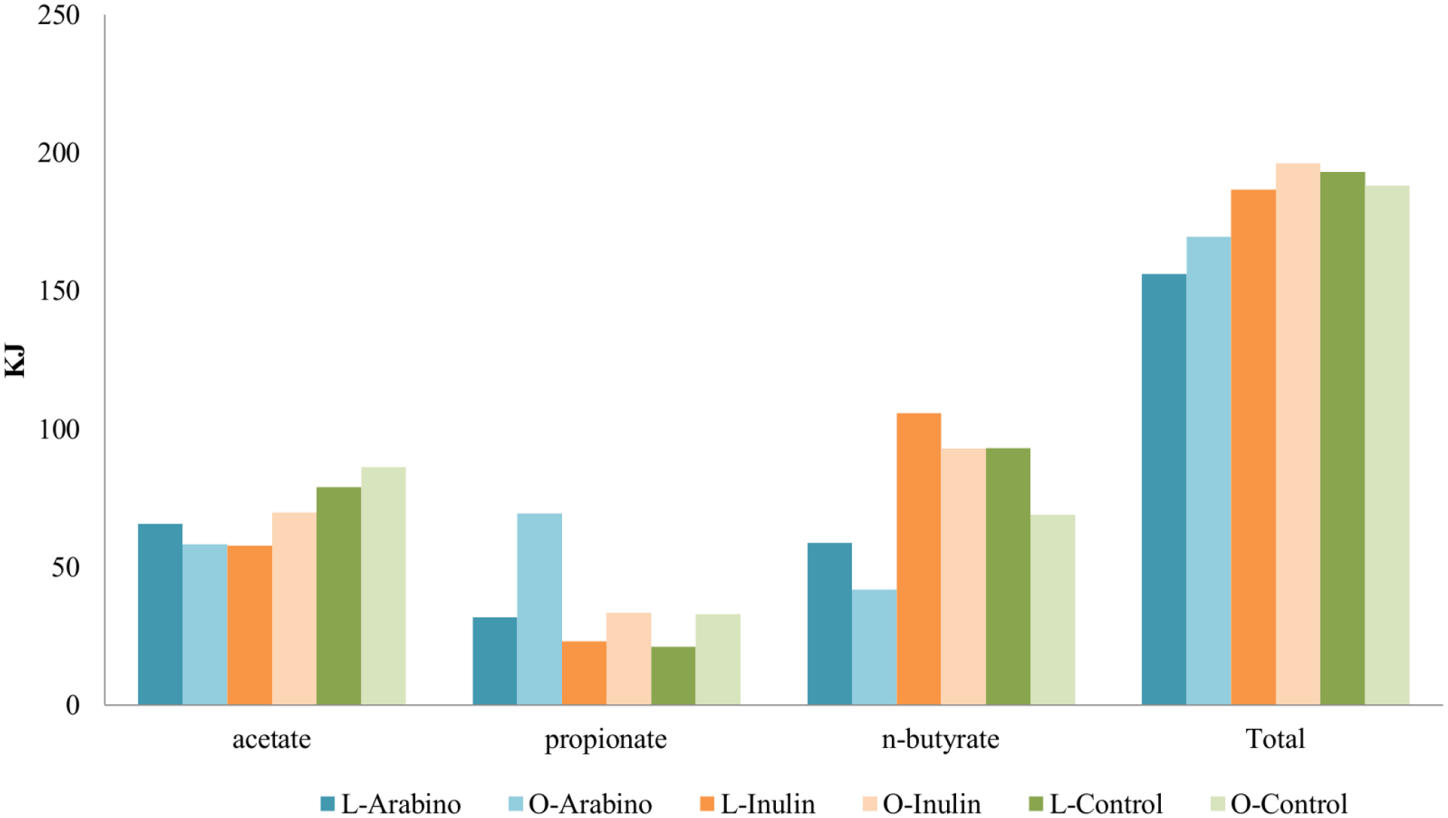
Energy extraction. Values for the individual SCFA and the sum (total) obtained after 72h fermentation experiments of AG, IN and control using lean or obese microbiota.

The microbiota from obese volunteers fermenting AG and IN extracted (slightly) more energy when compared to the lean fermentations, in accordance with the higher SCFA production. Controls remained quite similar with respect to energy extraction (Fig 2).

#### Compositional changes

By calculating L/O ratios (at the genus level), it was found that the lean microbiota had an increased relative abundance of *Faecalibacterium* (75 fold), *Dorea* (30 fold), *Roseburia* (6 fold), *Blautia* (3 fold) when compared to the obese microbiota (S2 Table; for more information about the starting inocula refer to S1 Fig).

The fermentation of AG and IN resulted in the growth or decrease of specific genera. Table 2 (AG) and Table 3 (IN) show the effects observed in the different microbiotas per substrate tested, compared to the control.

**Table 2:** Relative change of bacterial genera after 72h of fermentation experiments of arabinogalactan in TIM-2 compared to control.

	Arabinogalactan	
Genus	Lean	Obese
*Lactobacillus*	0.49	2.56
*Enterococcus*	0.51	1.65
*Unclassified*	0.99	2.24
*Escherichia*	1.11	0.98
*unclassified Clostridiaceae*	0.37	1.29
*unclassified Lactobacillaceae*	0.25	3.70
*unclassified Enterococcaceae*	0.55	1.39
*Shigella*	0.57	1.57
*Faecalibacterium*	0.35	1.53
*Bacteroides*	1.33	20.47
*Eubacterium*	0.54	2.95
*unclassified Enterobacteriaceae*	2.78	1.18
*unclassified Eubacteriaceae*	0.86	137.78
*Weissella*	74.12	0.00
*Collinsella*	1.63	0.01
*Pseudomonas*	0.88	1.34
*Blautia*	0.84	1.95
*Turicibacter*	0.21	1.58
*Dorea*	0.95	14.16
*unclassified Bacteroidaceae*	1.19	13.97
*Fusicatenibacter*	0.03	16.98
*Parabacteroides*	0.31	10.11
*unclassified Peptostreptococcaceae*	0.65	3.22

**Table 3:** Relative change of bacterial genera after 72h of fermentation experiments of inulin in TIM-2 compared to control.

	Inulin	
Genus	Lean	Obese
*Bifidobacterium*	0.77	1.96
*Lactobacillus*	0.15	1.08
*Unclassified*	0.83	2.57
*unclassified Bifidobacteriaceae*	1.09	1.83
*unclassified Lactobacillaceae*	0.15	1.57
*Faecalibacterium*	0.97	2.70
*Bacteroides*	2.84	0.89
*Collinsella*	1.10	0.36
*unclassified Ruminococcaceae*	0.98	3.27
*unclassified Coriobacteriaceae*	9.06	0.69
*Blautia*	0.49	4.53
*unclassified Bacteroidaceae*	2.62	0.88
*Fusicatenibacter*	0.21	27.99
*unclassified Lachnospiraceae*	0.73	2.50

Arabinogalactan. Genera that increased after the fermentation of AG by the obese microbiota but decreased in the experiments with the lean microbiota are the well-studied: *Lactobacillus* (3 vs 0.49 fold), *Dorea* (14 vs 0.95 fold), *Fusinibacter* (17 vs 0.03 fold), *Parabacteroides* (10 vs 0.31 fold), *Faecalibacterium* (2 vs 0.35 fold), and *Blautia* (2 vs 0.84 fold). At the species level the growth of *B*. *longum* was stimulated in the fermentation with the lean microbiota while it decreased in the obese (2 vs 0.21 fold, respectively; S3 Table) this effect was opposite for *L*. *mucosae* (0.50 vs 2 fold, respectively). When compared to the control, *B*. *caccae* and *B*. *thetaiotaomicron* notably increased in the lean microbiota (44 and 7 fold, respectively) whilst the growth of *L*. *gasseri* (12 fold) was stimulated in the fermentations with the obese microbiota.

Inulin. After the fermentation of IN by the obese microbiota an increase in *Bifidobacterium* (2 vs 0.8 fold), *Faecalibacterium* (3 vs 0.97 fold), *Blautia* (5 vs 0.5 fold) and *Fusicatenibacter* (28 vs 0.2 fold) was observed with respect to the lean microbiota. The fermentation of IN promoted the growth of *B*. *adolescentis* and unclassified *Bifidobacterium* in the obese microbiota when compared to lean (5 vs 0.66 fold; 2 vs 0.76 fold, respectively; S3 Table). *Enterococcus faecalis* was increased in the fermentations with the lean microbiota when compared to control (6 fold).

#### Modulatory effect of the substrates on activity and composition of the microbiota

Species in general were found to be divided in two groups: species that were positively correlated with *n*-butyrate (found in O-inulin and L-inulin experiments) and species that were negatively correlated to acetate, propionate and BCFA production (found in L-Inulin and L-arabino).

**Fig 3:**
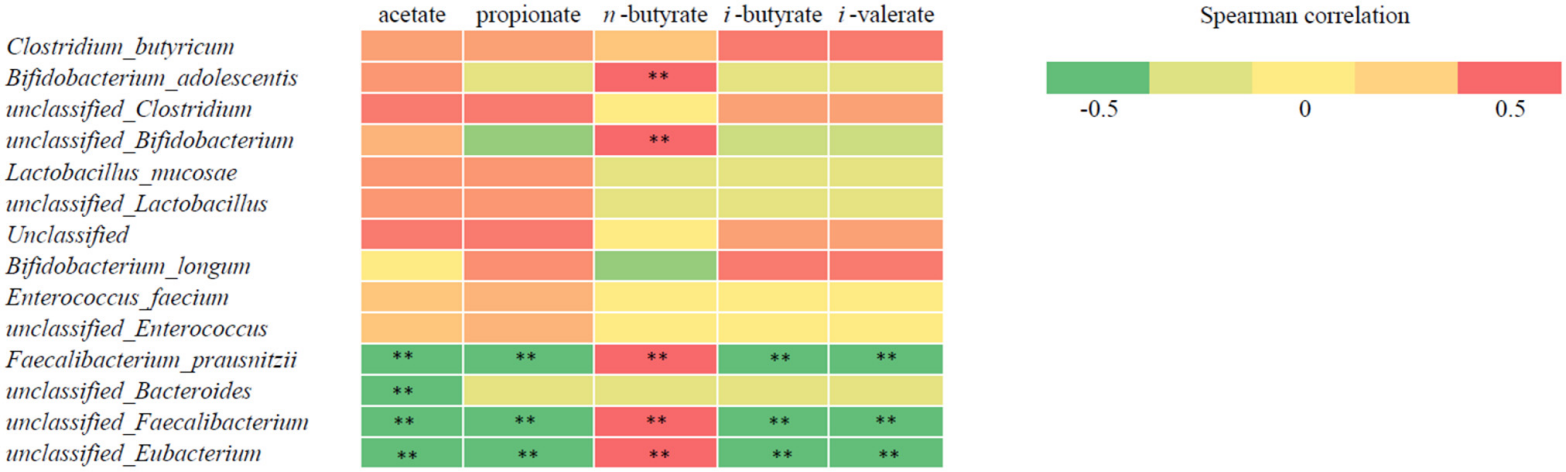
Correlation of metabolites and a subset of marker bacterial species. Rows correspond to bacterial species; columns correspond to measured metabolites. Red and green denote positive and negative correlation, respectively. The intensity of the colors represents the degree of association between taxa abundances and metabolites as measured by Spearman's correlations. ** indicate associations significant at the 0.01 level (2-tailed).

More specifically, correlation analysis performed showed a significant correlation of *B*. *adolescentis*, unclassified *Bifidobacterium*, *F*. *prausnitzii*, an unclassified *Faecalibacterium* and an unclassified *Eubacterium* with *n*-butyrate production; acetate, propionate and BCFA production were also significantly correlated with *F*. *prausnitzii* and unclassified *Faecalibacterium* but with an unclassified *Bacteroides* as well (Fig 3).

## Discussion

The addition of fiber to food products has been proposed to reduce the caloric density and glycemic impact of meals [36]. Therefore, there is growing interest in the use of functional fibers in the form of food ingredients, additives or supplements in order to fortify the Western diet without compromising the palatability of the food, especially in long-term weight management programs [36, 37].

Other properties of dietary fiber have been well documented and it has been observed that it exerts a wide array of biochemical, neurohormonal and microbiological effects in the human body [36]. Here the gut microbiota, as a metabolic organ, has been found to be influenced by fiber consumption.

The amount and type of dietary fibers consumed have a direct impact on the microbial fermentation capacity [38]. In this respect, studies have found that the gut microbiota from obese subjects could be more efficient in extracting energy from diet than lean subjects [11, 39, 40]. However, findings are controversial and it has been proposed that fermentation of fibers, and consequently their health effects, may be substrate dependent. Substrate dependency could be explained by the fact that dietary fibre reflects a heterogeneous group of compounds that differ in their chemical structure and physico-chemical properties, therefore reflecting on different physiological functions or health benefits [41, 42].

In this study, AG and IN were provided to the microbiota from lean or obese subjects and 72 h fermentation experiments were performed. The aim was to compare the differences in the fermentation profiles of these two fibers with respect to microbiota composition, but particularly with respect to production of the microbial metabolites SCFA, which are an energy source for the host.

Determination of fiber fermentation in humans and rats is a common approach to characterize the capacity of the gut microbiota to ferment a specific substrate. However, these studies are expensive, time consuming and in the case of rats the metabolic products yield from the fermentation of several types of fiber have been found to be significantly lower when compared to humans [43–45]. Moreover, studies in humans are limited because of the limited sampling capacity (non-invasively only feces can be collected), while most of the SCFA produced are taken up during transit of the chime through the colon (estimated at 95% of produced SCFA) [46]. Here, we used the TIM-2 system as an alternative tool to animal and human studies. *In vitro* systems simulating the large intestine have been validated and found to accurately predict the fermentation of fibers in human subjects by presenting the same magnitude of the differences in SCFA production [43]. In the current study pooling of fecal samples was performed to create a standardized microbiota which was subsequently frozen and stored. Both pooling [27] and the impact of freezing [28] have been validated before.

However, besides the lack of host interactions in such *in vitro* systems, another limitation is the characteristic variability of the microbiota used to inoculate the models, which is derived from a group of donors.

Involving different participants in a study like the present one constitutes one of the factors that influence the sometimes contradictory results in gut microbiota research. Still, the participants recruited for this study were considered according to their diet, consumption of prebiotics, probiotics and medication as specified in the M&M section. We considered including the microbiota from only those volunteers who fitted the best for the requirements from the two groups that we studied (i.e. lean and obese). Importantly, BMI index was prioritized given that the main goal of this investigation was exploring the differences between lean and microbiota fermenting AG and IN. We want to emphasize that the BMI index among subjects was very similar. This was reflected by the low standard deviation value observed in both groups (M&M). By setting the inclusion requirements mentioned above we believe that the outcome of our studies are the result of a lean or obese microbiome phenotype. Still, we acknowledge that the age difference from our group of volunteers might have contributed to differences in the microbiota.

The current study provides more evidence for the consumption of specific ingredients with the aim of modulating the gut microbiota in the context of obesity.

### Impact of the substrates on metabolic activity

When compared against each other and against the control, both fibers presented different fermentation kinetics (in terms of SCFA production) (Fig 1). The difference between the production of propionate and *n*-butyrate in AG experiments using both microbiotas (S2 Fig) is interesting in the light of the discussion of the impact of microbial metabolites in obesity. The increase in propionate production in the experiments with the obese microbiota suggests that via this metabolite AG could be protective against inflammation and promote satiety in obese subjects.

Despite that most studies about the anti-inflammatory role of SCFA have been focused on the effects of *n*-butyrate and acetate [47, 48], there is evidence pointing to propionate as a metabolite with a strong role against inflammation [49]. On the one hand, propionate acts as a ligand of G-protein-couple receptors (GPCR) 41 and 43 [50]. These receptors when activated, induce an increase of GLP-1 (which slows down gastric emptying and promotes satiety) and PYY (which up-regulates food digestion and absorption). Besides this, it has also been shown that when they are absent (at least for GPCR43 in knockout mice) there is an exacerbated inflammation in inflammatory-disease models [51]. Moreover, propionate has been found to decrease fatty acid levels in plasma [47]. As there is also evidence indicating that high plasma levels of fatty acids cause inflammation and consequently insulin resistance, the postulation of propionate as a molecule with anti-obesity properties is reinforced.

On the other hand, propionate has also been linked to autism [52] and hence, an increase in this metabolite may not be desirable, although the mechanism is not entirely clear.

In this study, IN was found to increase the production of *n*-butyrate in the fermentations with the lean microbiota (S2 Fig) when compared to the obese. The butyrogenic effect of IN has been previously observed *in vivo* and *in vitro* [16, 18, 53–57]. *n*-Butyrate has been postulated as a molecule with health benefits for the human host since it has been found to be an important source of energy for colonocytes, with a potential protective role against colon cancer. In addition, it has been inversely correlated with inflammatory bowel diseases such as Crohn’s disease [58–61]. To our knowledge only two studies have tested *in vitro* the fermentation of IN in both microbiotas (lean and obese). Both Sarbini *et al* [16] and Bussolo de Souza *et al* [18] found that the obese fermentation of IN produced higher concentrations of *n*-butyrate when compared to the lean fermentation. The inocula composition is not the same all the time (S2 Table) and that may have contributed to our results. Nevertheless, we have observed that there are some compositional similarities in both inocula despite being prepared in different years (S2 Table).

The fermentation of AG showed decreased BCFA concentrations in the fermentations with the obese microbiota when compared the lean. This supports the work from Vince and colleagues [62] and Robinson & Slavin [20] who observed a significant decreased in products from proteolytic fermentation (specifically ammonia) by intestinal bacteria after the supplementation of AG. However, our study brings evidence, for the first time, about this beneficial effect of AG in obese subjects.

Though production of BCFA was not lower in the obese microbiota fermenting IN when compared to lean, at least, when it is compared to control, the fermentation of IN with the lean microbiota is lower, giving also a good indication of the prebiotic effect of IN in ameliorating proteolytic fermentation. This effect has been previously observed *in vitro* as well as *in vivo* [29, 63, 64].

### Energy extraction

The hypothesis that the gut microbiota in obese individuals facilitates the additional extraction of calories from diet has been previously reviewed [38, 65, 66]. In this study we were not able to confirm that the energy yield (in terms of SCFA produced) was higher in the feces from the obese donors in comparison with the lean donors (S1 Table). However, the absorption of SCFA has been described as a very efficient process since only 5–10% is excreted in the feces [67]. Furthermore, in a study performed by Jumpertz and colleagues [68] overfeeding in lean subjects was associated with a greater decrease in stool energy loss showing a relation between loss of energy in feces and energy load. Therefore, based on our results we could hypothesize that obese individuals may have higher capacity of absorbing SCFA in their gut and, therefore, may have lower amounts of these metabolites in their feces compared with lean subjects.

However, we found a substrate dependent effect on the metabolic activity and consequently energy extraction when fermentation of both AG and IN were performed. These findings confirm our previous observations where a higher amount of energy extracted from the fermentation of IN was also found after fermentation by obese microbiota when compared to lean [18]. Although the difference is small, over a prolonged period this may add up to several kilos of body weight, since, as previously mentioned, an elevated production of SCFA contributes to a higher energy input to the host. But at the same time these metabolites have also been found to present satiety-enhancing properties via the activation of GPCRs. Thus, it could be that the enhancement of the production of these metabolites could be protective against obesity in this population, despite their energy content. Still, more research is needed in this area.

### Compositional changes

When the inocula prepared from both lean and obese subjects (previous study; 2012) is compared to the inocula from the present study (2014), we observed some compositional differences in the shared bacterial groups. This shows that not all inocula are the same. However, there are some similarities in some increased groups observed from the L/O ratios (S2 Table) making it possible to distinguish certain bacteria belonging for example to *Gemmiger*, *Dorea*, *Roseburia*, *Alistipes* genera which both times were highly abundant in the lean microbiota and deserve being more investigated about their potential role in leanness.

After fermenting AG by the different microbiota the growth of some groups of bacteria was highly stimulated in one microbiota whilst they decreased in the other. Between the groups that were benefited from the fermentation of AG in the obese microbiota we found *Faecalibacterium*, *Dorea* and *Blautia*. Judging from the ratios calculated in order to compare both inocula (L/O 2014; S2 Table), it seems that the community from obese donors tend to re-structure towards the microbiota from lean donors after the fermentation of AG (Table 2).

This effect was also previously observed by Bussolo de Souza *et al* [18] and Condezo-Hoyos *et al* [19] when testing the prebiotic effects of cassava bagasse and different apple cultivars on the composition from lean and obese microbiota.

*Lactobacillus* was observed to increase in the obese microbiota fermenting AG (Table 2). Furthermore, we also described an increase of the species *L*. *mucosae* and *L*.*gasseri* in the obese microbiota after fermenting AG. Our results are in agreement with Robinson & Slavin [20] when showing that a diet supplemented with AG increased the concentration of *Lactobacillus* in the feces from healthy participants. In addition, a study conducted by Santacruz *et al* [69] showed a parallel reduction of body mass index (BMI) and increase of *Lactobacillus* spp. concentrations in obese adolescents suggesting a potential role of this genera in obesity and body weight control. Based on these findings we suggest that our study brings more evidence about how a prebiotic such as AG can beneficially influence the composition of the microbiota from obese subjects in weight management.

The lean microbiota fermenting AG presented an increase in *B*. *thetaiotaomicron*. The growth of this species on AG has also been found by others [70]. *B*. *thetaiotaomicron* colonization has been observed to elicit gene expression involved with the fortification of the intestinal barrier function and the maintenance of mucosal integrity which may suggest to be specially beneficial in obese subjects [71].

The obese microbiota composition on IN shifted the simulated gut environment into a more healthy milieu with increase of beneficial bacteria belonging to the *Faecalibacterium*, *Blautia*, *Fusicatenibacterium* and *Bifidobacterium* genera. The bifidogenic effect of IN was demonstrated to be more pronounced in the obese microbiota when compared to lean (Table 3). Interestingly, this effect was shown to selectively stimulate the growth of *B*. *adolescentis* (S4 Table). This is in agreement with the upregulation *of B*. *adolescentis* by IN also found by Ramirez-Farias *et al* [22] and *B*.*animalis* by Venema and Maathuis [72]. In fact the latter authors hypothesized that within the *Bifidobacterium* genus, the diversity was diminished by inulin. This was also observed to some degree in our experiments (S4 Table).

The difference in the bifidogenic effect of IN in the obese microbiota, especially in the specific case of *B*. *adolescentis* growth, can be explained by i) a long-known inverse relation between initial amounts present and the observed increase [73]. As observed e.g. by Korpela *et al* [74] and other authors, the lower the abundance of *Bifidobacterium spp*. at the starting point of an intervention, the more the increase observed after the administration of an specific prebiotic, and vice versa [74–76]; or ii) the high specificity of IN for stimulating the growth of certain bifidobacterial species as observed by Venema & Maathuis [72]. A high abundance of bifidobacteria could be protective in obesity since it is speculated that this bacteria may decrease pro-inflammatory cytokines and decrease endotoxaemia which can improve glucose-induced insulin and glucose tolerance [16, 77].

## Conclusion

First line strategies to combat obesity include exercise and/or a balanced dietary regime. Though apparently simple, such changes in people’s life are difficult to maintain and in most of the cases patients struggle to follow these recommendations. Due to the complexity of this condition, it is vital to identify weight loss methods by which subjects can successfully achieve long-term results. In this respect, using fibers is a potential tool to supplement diet in weight management due to their satiety aspects, as well as in modulating the gut microbiota. Here in this study we have identified the potential of arabinogalactan and inulin in stimulating a gut community more related to a lean profile. Metabolically, arabinogalactan fermentation showed a higher production of propionate when compared to *n*-butyrate in the obese microbiota fermentations. In general, lean microbiota produced more *n*-butyrate from the fermentation of both substrates when compared to the obese microbiota. This would be interesting to investigate in light of the potential activation of different GPCRs by these SCFA. However, these effects should be more studied in humans focusing especially in the role of these fibers in satiety.

## Supporting Information

S1 Fig Abundance (%) of the major groups of genera in the microbiota from lean and obese subjects. At genus level, it was observed that differences among the inocula are driven by the abundances within the same groups of bacteria (S1 Fig). The major groups in the lean microbiota were *Bifidobacterium*, *Clostridium*, *Lactobacillus* and *Enterococcus* (23, 34, 14 and 13%, respectively) and in the obese microbiota were *Clostridium*, *Lactobacillus*, *Enterococcus*, *Bifidobacterium* (46, 17, 15 and 10%). Analysis of the species found in the inoculum (t0) suggests that the lean microbiota has a more diverse population of *Bifidobacterium* which included *B*. *adolescentis*, *B*. *longum* and an unclassified group (7, 4 and 8%, respectively) whilst the obese microbiota mainly contains *B*. *longum* and an unclassified group (6 and 3%, respectively). Both microbiotas share a high abundance of *C*. *butyricum* (lean: 23%; obese: 30%).
(TIF)

S2 Fig SCFA ratios (%) from the different diets at t72. (TIF)

S1 Table Metabolites and energy extraction (in terms of SCFA) measured in the feces from the volunteers. (XLSX)

S2 Table Ratio L/O. Relative ratio of bacterial genera different between lean and obese at t0 in TIM-2.
(XLSX)

S3 Table Relative change of bacterial species after 72h of fermentation experiments of arabinogalactan and inulin in TIM-2 using microbiota from lean and obese individuals. (XLSX)

S4 Table Bifidobacterium species abundance from the fermentation of inulin. In both cases (L and O-fermentations) *B*.*adolescentis* dominated the Bifido species after 72 h. In lean microbiota a complete domination is observed while in the obese microbiota it is about 25 fold higher (whereas at the start it was only ~8%). Previous testing of the *in vitro* effects of IN fermentation in both microbiotas (lean and obese) found that the obese fermentation of IN produced higher concentrations of *n*-butyrate when compared to the lean fermentation [16, 18]. The difference with our study could be explained by the different stimulation of certain butyrogenic strains which in this particular case found more favourable to growth in the lean microbiota as observed in S3 and S4 Tables.
(XLSX)
