# Supplementary figures and images for: The Gut Microbiota from Lean and Obese Subjects Contribute Differently to the Fermentation of Arabinogalactan and Inulin
Source: PLoS One. 2016 Jul 13;11(7):e0159236. doi: 10.1371/journal.pone.0159236 (PMC4943740; doi:10.1371/journal.pone.0159236)

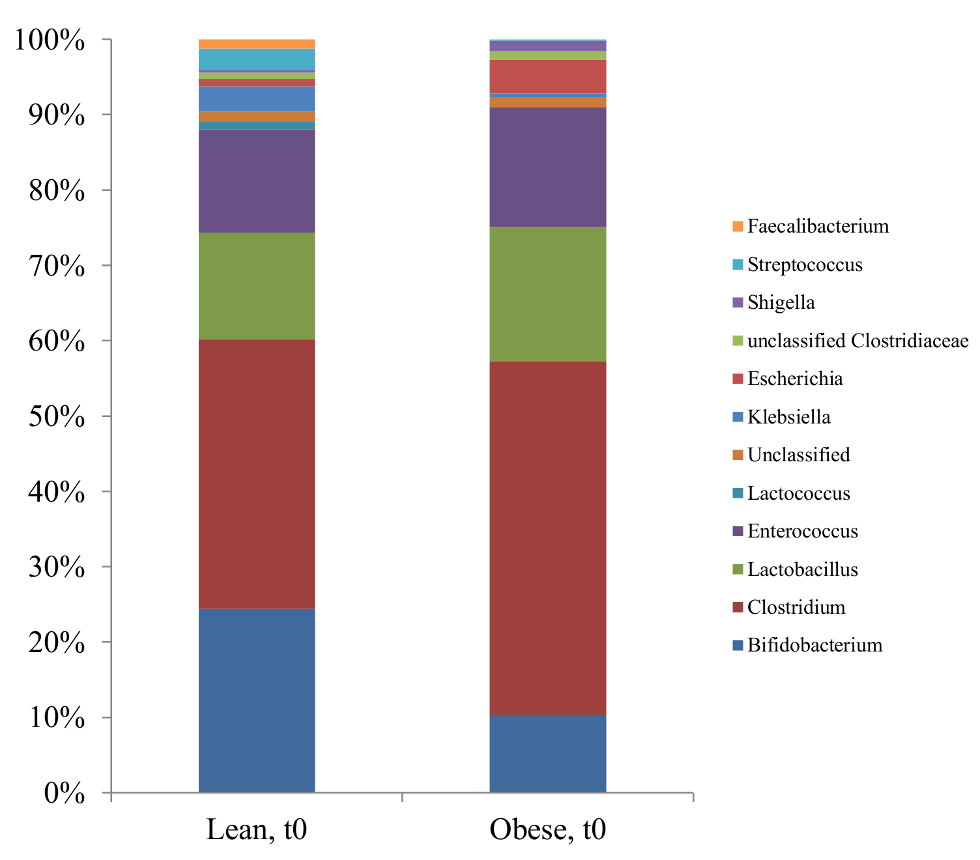

Supplement: S1 Fig — At genus level, it was observed that differences among the inocula are driven by the abundances within the same groups of bacteria (S1 Fig). The major groups in the lean microbiota were Bifidobacterium, Clostridium, Lactobacillus and Enterococcus (23, 34, 14 and 13%, respectively) and in the obese microbiota were Clostridium, Lactobacillus, Enterococcus, Bifidobacterium (46, 17, 15 and 10%). Analysis of the species found in the inoculum (t0) suggests that the lean microbiota has a more diverse population of Bifidobacterium which included B. adolescentis, B. longum and an unclassified group (7, 4 and 8%, respectively) whilst the obese microbiota mainly contains B. longum and an unclassified group (6 and 3%, respectively). Both microbiotas share a high abundance of C. butyricum (lean: 23%; obese: 30%). (TIF) [file pone.0159236.s001.tif]

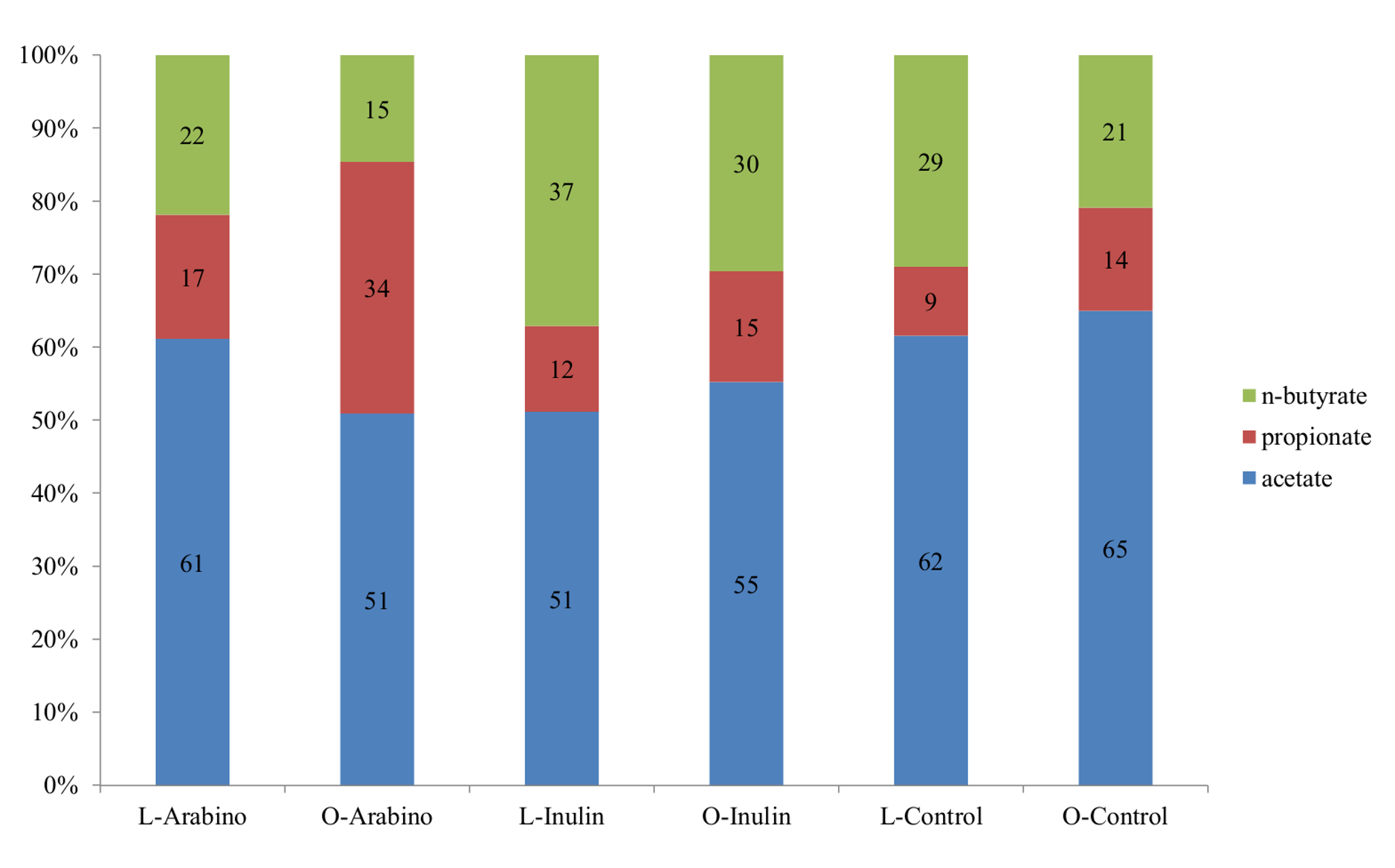

Supplement: S2 Fig — (TIF) [file pone.0159236.s002.tif]
